# Surface Aneurysmal Bone Cyst

**DOI:** 10.5334/jbr-btr.961

**Published:** 2015-12-30

**Authors:** Arn Van Royen, Filip Vanhoenacker, Jeoffrey De Roeck

**Affiliations:** 1 AZ Sint-Maarten Duffel-Mechelen/UZ Brussel, BE; 2 AZ Sint-Maarten and University (Hospital) Antwerp/Ghent, BE; 3 AZ Herentals, BE

**Keywords:** Aneurysmal Bone Cyst, Bone tumor, Fluid-fluid levels, Radiography, MRI

A 19-year-old boy presented with progressive pain since 6 month at the left thigh, aggravating during soccer and swimming.

Plain radiograph showed an osteolytic lesion with sclerotic margin (Fig. A, black arrow) and interrupted lamellar periosteal reaction (Fig. A, white arrow) at the posterolateral aspect of the diaphysis of the left proximal femur. Magnetic resonance imaging (MRI) was performed for further characterization of the lesion.

**Figure A:**
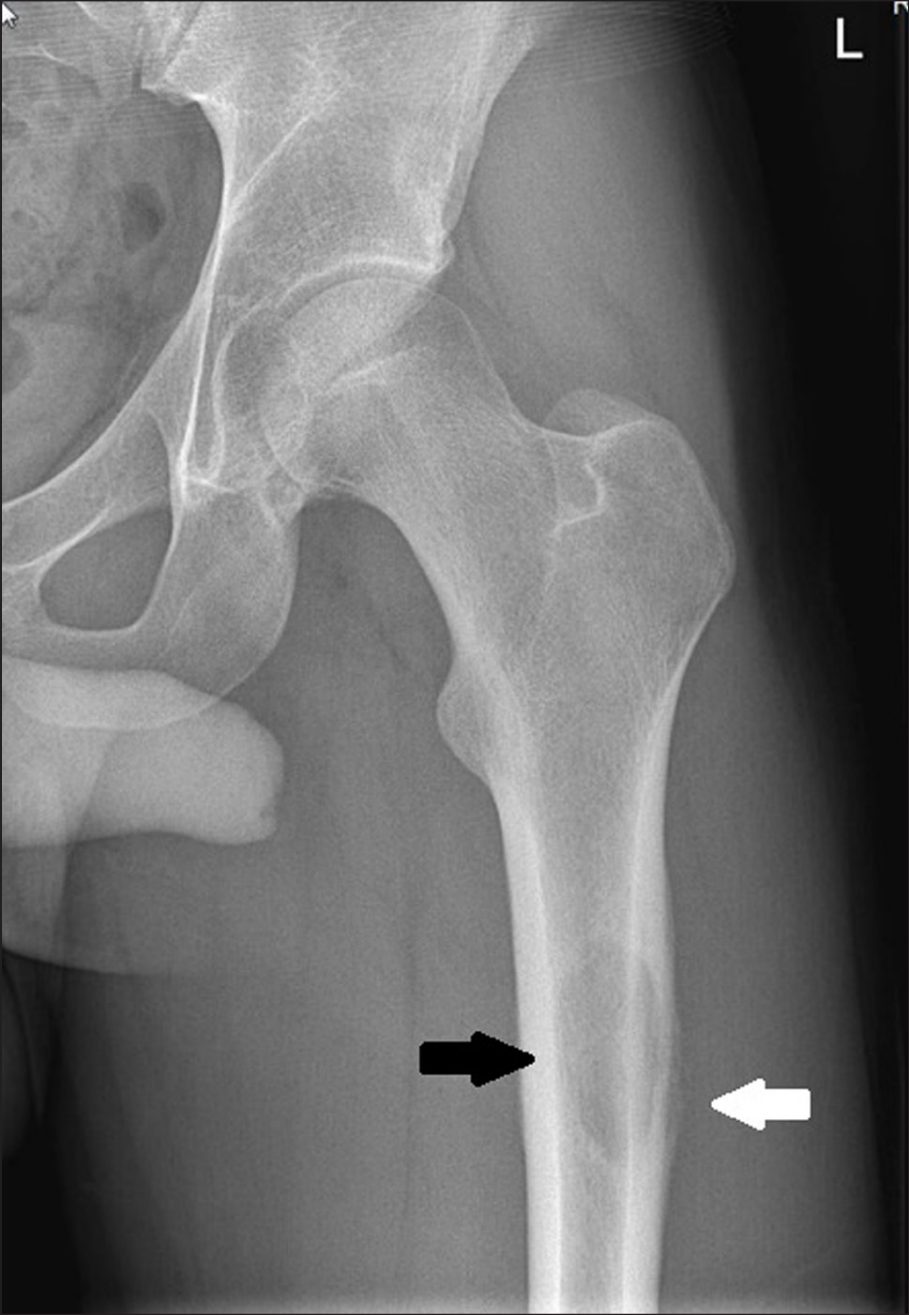


T2 weighted images (WI) showed a cortical bone defect and an interrupted shell-like periosteal reaction (Fig. B, white arrow) containing a fluid-fluid level (FFL) (Fig. B, black arrow) at the posterior aspect of the left proximal femur shaft. Contrast-enhanced T1-WI showed a multi-cystic appearance with contrast-enhancing cyst walls (Fig. C, black arrow). There was also bone marrow oedema within the femur and contrast-enhancing soft tissue changes adjacent to the femoral cortex (Fig. C, white arrow).

**Figure B:**
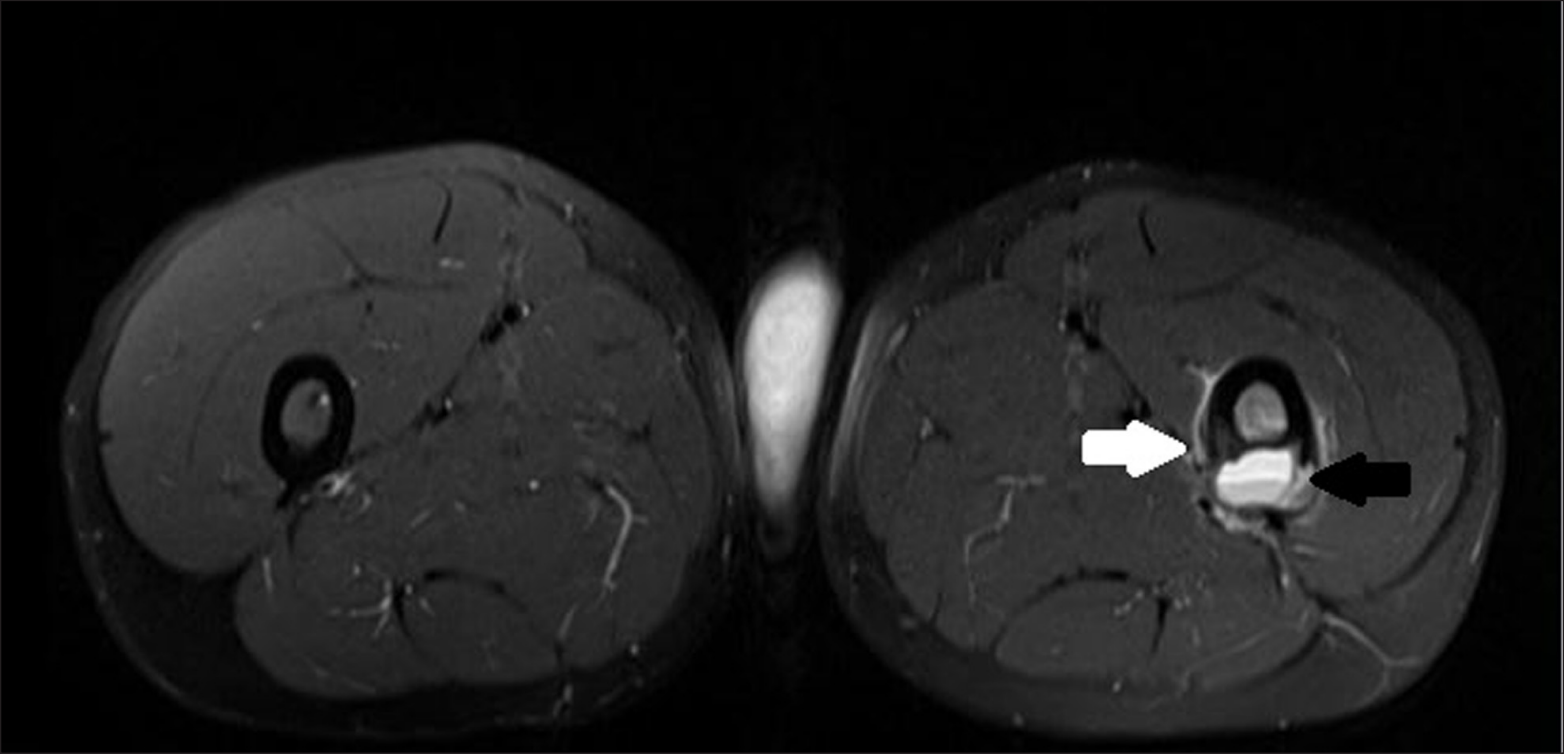


**Figure C:**
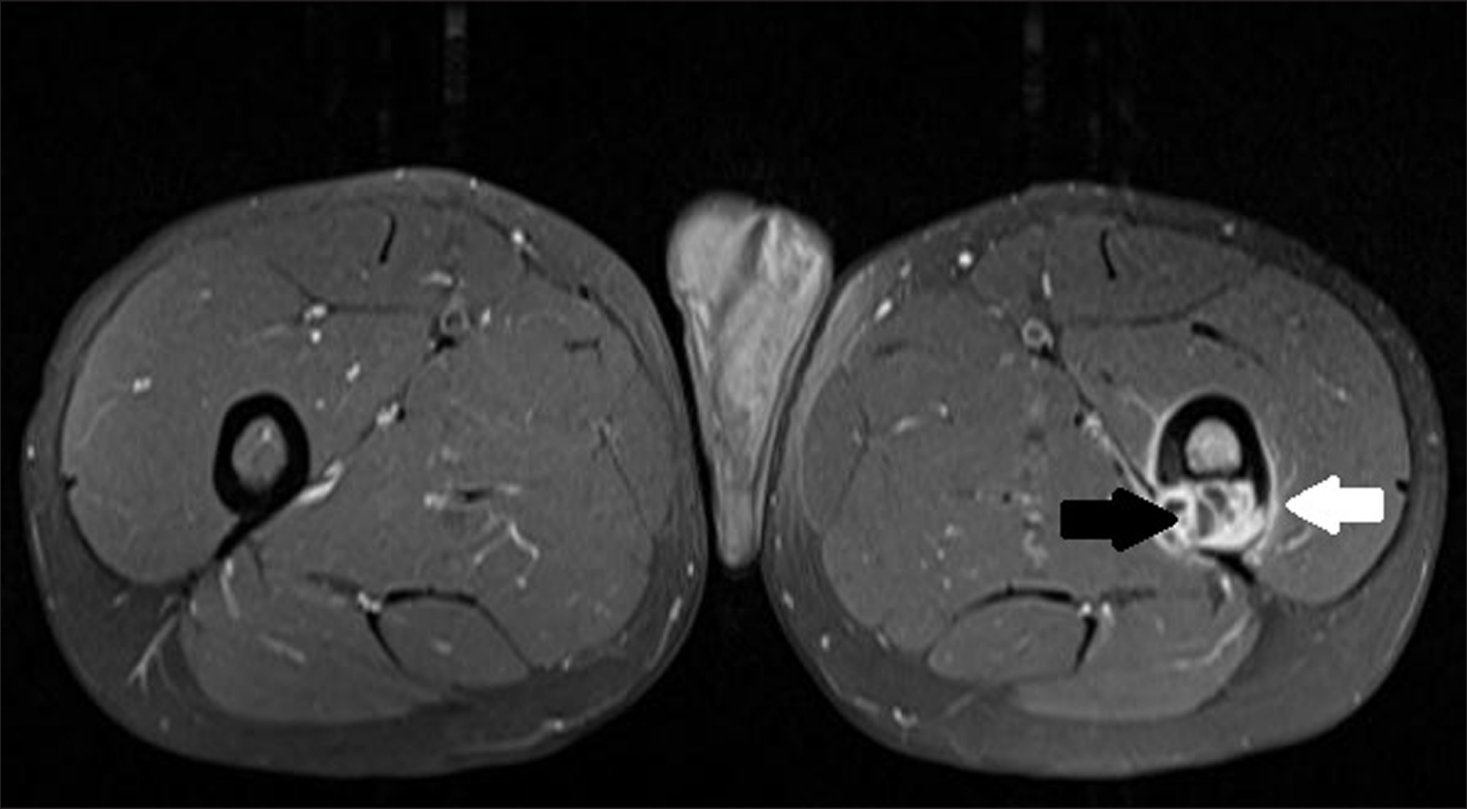


Because of the aggressive periosteal reaction, the patient underwent a biopsy and the final diagnosis of a subperiosteal aneurysmal bone cyst (ABC) was made. Subsequently, the patient was treated with curettage of the lesion.

## Comment

Aneurysmal bone cysts (ABC) are benign, haemorrhagic, expansile, osteolytic lesions composed of different blood-filled channels. ABC either arises de novo (primary ABC) or as a complication of trauma or underlying neoplastic disease (secondary ABC). ABC comprise between 1–2% of all bone tumours and in up to 90% of the cases patients are under the age of 20, mainly affecting the metaphysis of the long bones followed by the spine and pelvis. Most ABC are located intramedullary (80%), but more rarely they are localized at the surface, either subperiosteally or cortically. Although intramedullary ABC are typically confined to the metaphysis of the long bones, involvement of the diaphysis is not unusual in surface ABC.

Surface ABC may have irregular margins and are often associated with periosteal reaction mimicking an aggressive lesion on plain radiographs.

MRI is not only helpful in evaluating local extent but also for lesion characterization by demonstrating FFL and contrast-enhancing walls. Extent of fluid-fluid levels may be particularly helpful in differentiating ABC from a malignant lesion causing FFL (e.g. teleangiectatic osteosarcoma). If FFL is seen in more than 2/3 of the lesion volume, it usually represent a benign ABC.

Differential diagnosis of a surface ABC include benign surface lesions such as subperiosteal hemangioma or haematoma, subperiosteal giant cell granuloma and periosteal chondroma. Malignant tumours such as teleangiectatic osteosarcoma should also be excluded.[1]

Treatment options consist of wide excision, curettage, sclerotherapy and grafting.

## Competing Interests

The authors declare that they have no competing interests.

## References

[1] Woertler K, Brinkschmidt C (2002). Imaging features of subperiosteal aneurysmal bone
cyst. Acta Radiol.

